# Changes in body composition, bone metabolism, strength, and skill-specific performance resulting from 16-weeks of HIFT

**DOI:** 10.1371/journal.pone.0198324

**Published:** 2018-06-15

**Authors:** Yuri Feito, Wade Hoffstetter, Paul Serafini, Gerald Mangine

**Affiliations:** Department of Exercise Science & Sport Management, Kennesaw State University, Kennesaw, GA, United States of America; University of Rome, ITALY

## Abstract

High Intensity Functional Training (HIFT) is a training modality, characterized by multimodal exercises performed at high-intensity. Little is known about the training adaptations that occur as a prolonged training program. The purpose of this study was to examine changes in body composition, bone metabolism, strength, and skill-specific performance over 16-weeks of HIFT. Twenty-six recreationally active adult males (n = 9; 34.2 ± 9.1 y; 91.5 ± 17.7 kg; 178.5 ± 5.4 cm) and females (n = 17 = 36.4 ± 7.9 y; 91.5 ± 17.7 kg; 162.9 ± 7.0 cm) completed pre and post training assessments of body composition (Dual-Energy X-Ray Absorptiometry) and performance measures. Performance was assessed using three HIFT workouts (WOD 1–3) to assess strength, skill, and metabolic performance. Aside from the body composition measurements, all assessments were carried out at the local training facility. Training included participation in HIFT a minimum of twice a week for 16-weeks. Repeated measures analysis of variance revealed a significant gender x time interaction in Bone Mineral Content (BMC) (p = 0.027), where improvements favored women (1.0% ± 1.1%, p = 0.004) over men (-0.1% + 0.8%, p = 0.625). Further, region-specific analysis indicated that women (2.5% ± 3.0%, p < 0.005) experienced greater improvements in the trunk compared to men (-0.3% ± 1.8%, p = 0.621), while changes in leg BMC were comparable between women (0.8% ± 1.0%, p < 0.001) and men (0.3% ± 0.6%, p < 0.001). Although no other interactions were observed, significant performance improvements were noted for all participants in WOD 1 (18.3% ± 16.8%), absolute 5RM (14.4% ± 9.7%), relative 5RM (15.4% ± 9.2%), WOD 2 (5.7% ± 6.5%), and WOD 3 (–17.3% ± 14.7%). These data indicate that 16-weeks of HIFT resulted in positive outcomes in strength, metabolic conditioning performance, and body composition.

## Introduction

Resistance training is known to stimulate improvements in body composition, skeletal tissue, and muscular strength and endurance [1, 2]. Despite its benefits, lack of time and motivation are often cited as the main factors that make compliance to traditional exercise programs difficult [3, 4]. However, greater success has been reported when exercise is performed under the supervision of a certified professional, while those without qualified supervision have been reported to utilize insufficient training loads to stimulate potential adaptations [5, 6]. Another common practice within the fitness industry has been to combine several training modalities into a single regimen (e.g., cross-training) to obtain multiple training benefits within the same time frame. Recently, a unique version of this concept, termed “high-intensity functional training” (HIFT; e.g., CrossFit^®^ training) has grown in popularity. It includes a combination of exercise modes (e.g., resistance training, gymnastics, and aerobic conditioning) that are performed at a high-intensity while in a group environment and led by a certified and trained professional [7].

Though limited, evidence suggests that HIFT may have several physiological benefits in healthy adults [7–10]. Buckley et al. [8] reported improvements in aerobic and anaerobic performance, and muscular strength and endurance in recreationally-trained females after a six-week HIFT protocol. Likewise, muscular strength and endurance, as well as aerobic capacity and flexibility were improved following 8-weeks of HIFT in active military personnel [11]. Aerobic capacity improvements have also been reported following HIFT in adult cancer survivors and healthy adults, in addition to enhancements in body composition [9, 10]. However, little is known regarding the impact of HIFT on adaptations to lean tissue (i.e., bone mass and bone-free lean mass).

Even though pharmacological agents that enhance bone remodeling are available to individuals with decreased bone mass [12], research has shown that physical activity is the only intervention able to provide improvements in body mass and strength, while reducing the risk of falls [13]. Therefore, the American College of Sports Medicine (ACSM) suggests that adults who participate in resistance training (and other load-bearing exercises) possess higher bone mineral density (BMD) than adults who were engaged in normal physical activity (i.e. walking, cycling) [13]. In addition, research has established that competitive weightlifters and powerlifters tend to show superior BMD when compared to other athletes and sedentary individuals [14–17]. The heavier resistance loads (≥ 60% of one repetition maximum [1-RM]) typically employed by these athletes are believed to be essential for stimulating skeletal remodeling [1].

In 2008, Ratamess and colleagues demonstrated how women who did not utilize a certified fitness professional reported utilizing lower resistance loads during resistance training (38–48% 1RM) compared to those who employed a trainer’s services (43–57.4% 1RM) [5]. While all of the women in that study reported using machines for resistance exercise, a lesser percentage of those women who did not employ a trainer reported using free weights and dumbbells in addition to machines during their exercise routines [5]. Machines alone may not be adequate for stimulating skeletal adaptations because most are performed from a seated position and would not load the spine [1]. In contrast, HIFT incorporates free weights for a variety of multi-joint, spinal loading exercises that are often prescribed at intensity loads that exceed 60% of 1RM. Thus, we contend that regular participation in HIFT could significantly augment strength and skeletal mass (i.e., BMD and bone mineral content [BMC]) in women. Indeed, a recent analysis of the top 1500 female CrossFit athletes indicated that these athletes could lift on average 1.04–2.35 times their body mass (67–158 kg) in the squat, deadlift, clean & jerk, and snatch exercises [18]. Although their actual training regimen is unknown, these values are consistent with those previously reported in women who incorporate higher intensity loads into their training [19]. Nevertheless, it remains unclear whether skeletal mass and strength would improve in recreationally-active adults who participate in HIFT. Additionally, Caserotti and colleagues [20] reported on the effects of explosive-type heavy-resistance training (75–80% of 1 repetition maximum) twice-per-week, for 12-weeks in women between 60 and 65-years old and over 80-years old. Investigators demonstrated improvements in rate of force development (21% vs. 51%, respectively) and maximal voluntary contractions (18% vs. 28%, respectively) after 12-weeks of training suggesting that heavy-resistance training is well tolerated among women even later in life, which could provide independence and reduce risks of falls and disability, as a result of type II muscle fiber hypertrophy [21].

The general frequency recommendation for healthy adults is to train the entire body at least 2–3 days per week [1, 22], with more frequent sessions becoming useful in advanced lifters and specialized training (e.g., greater exercise selection and volume per muscle group in accordance with more specific goals). Failure to train specific muscle groups with sufficient frequency might negatively impact the intended adaptations [22]. Unlike traditional training programs that typically employ systematic variation of the same full-body or split routine throughout an entire training cycle to evenly target specific muscle groups [22, 23], HIFT strives to maintain constant variation within each microcycle to promote general physical preparedness (i.e., the simultaneous development of aerobic and anaerobic bioenergetic systems or “metabolic conditioning”) [24]. Consequently, an even distribution of targeted muscle groups may not occur during each week of training. This could be problematic because the musculature of the upper- and lower limbs have been observed to respond differently to exercise-induced muscle damage [25] and ultimately adapt at different rates [26]. Thus, it is possible that musculoskeletal adaptations to HIFT may vary by region.

Though physiological and performance adaptations are often used as benchmark measures of progress, traditional measures alone may not be sufficient to monitor HIFT. Several studies have examined the relationship between skill-specific performance, and resistance training outcomes in adults and suggest that experience [27], and whole-body strength [28], are the best predictors of performance in this type of training modality. Likewise, Serafini and colleagues recently reported that clear differences in strength, power, and sport-specific skill exist between various tiers of competitive rank in athletes participating in a form of HIFT [18]. However, Eather and colleagues [29] evaluated an 8-week, teenager-focused, HIFT program in comparison to traditional physical education and sports/leisure activities in 96 high school students. Although the authors observed significant improvements in several health-related fitness variables (i.e. waist circumference, body mass index, and sit-and reach), HIFT did not offer any greater advantages for developing resistance training skill competency except in push-ups. [29].

Considering that the focus of training varies daily during HIFT, it is unknown whether physiological and sport-specific skill adaptations simultaneously occur. Therefore, the purpose of this study was to examine the effect of 16-weeks of HIFT training on body composition, bone remodeling, as well as skill-specific performance among a group of relatively-active adults. We hypothesized that this type of training would elicit significant decreases in total and regional body fat, while improving lean and skeletal mass. We further hypothesized that sport-specific performance would improve following the 16-wk HIFT program.

## Materials and methods

### Study design

We conducted a 16-week prospective cohort study, using a convenience sample of recreationally active adults. Within two-weeks of beginning the 16-week HIFT intervention (PRE), anthropometric assessments (i.e., body composition and bone mineral characteristics) were completed in the human performance laboratory with participants having avoided food and any beverage other than water for four hours, exercise for 12 hours, and alcohol for 24 hours to complete. All performance assessments (i.e., strength, conditioning, and skill) were completed on three separate occasions during the first week of training at the local training facility. During performance measures, all participants were encouraged to eat a small meal, or snack two-to-three hours prior to performance testing sessions.

Subsequently, all participants completed each training sessions of the intervention at the same training facility and were asked to adhere to all staff instructions and participate in at least two training sessions per week. All training sessions and workouts were designed and directly supervised by at least one certified and trained instructor assigned by the training facility. The investigation was intended so that investigators did not have any control of the programming and design of the exercise program, while the staff at the training facility was not provided access to any of the participant’s data. Within two-weeks of finishing the 16-week program (POST), all PRE-assessments were repeated.

### Participants

Fifty-three recreationally-active adults with over three-months of experience were enrolled into this investigation. Following an explanation of all procedures, risks, and benefits, each participant provided his or her written informed consent to participate in the study. All participants were free of any known contraindications to moderate or vigorous exercise [30], and did not have any musculoskeletal, cardiovascular, pulmonary, or metabolic conditions that limited their ability to exercise; females who were pregnant were also excluded from this investigation. The Institutional Review Board at Kennesaw State University approved all procedures and study protocols prior to participant enrollment (Study #15–005).

### Anthropometric assessments

All anthropometric and body composition measures were completed prior to the performance measures. Height (cm) and body mass (kg) were determined using a stadiometer and an electronic physicians scale (Tanita WB 3000, Arlington Heights, IL) with the participants standing barefoot, with their feet together, and in light and comfortable clothing (e.g. shorts and t-shirt). Total body composition, bone mineral characteristics [bone mineral density (BMD) and bone mineral content (BMC)], and the regional estimates were determined via dual-energy X-ray absorptiometry (DXA) (Lunar iDXA, General Electric Healthcare, Madison, WI). Total body estimates of percent fat (%FAT), BMC (± 0.1 kg), BMD (± 0.1 kg · cm^-2^), and non-bone lean mass (NBLM; ± 0.1 kg) were determined using the company’s recommended procedures and supplied algorithms. Regional estimates of BMC, BMD, and NBLM were calculated by summing (BMC and NBLM) or averaging (BMD) values obtained for both arms (ARM), both legs (LEGS), and the spine and pelvis (TRNK) by following manual demarcations for these regions of interest. Quality assurance was assessed by daily calibrations performed prior to all scans using a calibration block provided by the manufacturer. All DXA measurements were performed by the same investigators using standardized subject positioning procedures. These methodology for obtaining values for these specific regions of interest had been previously determined to be reliable (ICC’s > 0.94) using a random subset of 10 healthy adults from the study population (25.1 ± 2.4 y; 81.1 ± 18.5 kg; 175.7 ± 6.8 cm).

### Performance assessments

Three separate workouts were used to assess performance. These workouts were designed by the training staff as part of an intra-facility “fitness challenge” and consisted of exercises that were common to HIFT and could be performed by all members. Briefly, all participants were encouraged to arrive to the training facility 10–15 minutes prior to commencing the group session. Prior to testing, all participants completed a 20-minute active warm-up period lead by an instructor and included jogging, calisthenics, and workout-specific exercises. Although intensity was not actually measured during the warm up period, participants were encouraged to maintain a light to moderate intensity and focus on working through an entire range of motion rather than at high intensity.

The first workout (WOD-1) was skill-based and required participants to complete 20 repetitions as fast as possible while maintaining proper technique for the squat-press exercise (i.e. thruster), followed by as many repetitions as possible (AMRAP) within a 2- or 3-minute period for three HIFT specific movements–double-unders, kettle bell swings, and burpees (Table 1). Since the time and number of repetitions varied for each movement, performance during each portion of WOD-1 was converted into a rate (i.e., repetitions per minute). Rates for each exercise during WOD-1 were averaged to reach a final performance score. The second workout (WOD-2) consisted of a combination of strength and conditioning movements. For the strength portion, participants were given 5–10 minutes to complete warm-up sets before attempting to determine their five-repetition-maximum (5-RM) in the front squat exercise. Subsequently, participants were allotted a 5-minute rest period before completing a 15-minute AMRAP consisting of a 350-meter row and 15 burpees. Participants were scored on the absolute (in Kg) and relative (kg · body mass^-1^) loads lifted during the 5-RM front squat, and they were also scored on the total number of repetitions completed during the AMRAP. Workout three (WOD-3) was a metabolic conditioning (METCON) challenge that required participants to complete a circuit of deadlifts, wall ball shots to a 9- and 10-foot target, for females and males, respectively, and sit-ups using a descending repetition scheme (i.e., 21, 15, 9, 6, and 3 repetitions) as fast as possible, while maintaining proper form of all movements. Time to completion (in minutes) was used to rate performance. WOD 1–3 were all completed on different days during the first week of the study. For WOD-1 and WOD-3 at POST, participants utilized the same resistance loads as PRE. Standardized technique and scoring were ensured by the training facility’s staff, not the investigators. Table 1 provides a detailed description of the three workouts used to assess progress in this challenge.

**Table 1:** Description of performance-based workouts.

Workout # 1 (Skill-Based)			
Movement	Load	Type	Scoring^
Thrusters	W: 34 kg—M: 52 kg	20 reps for time	Time to completion
Double-unders	Body Weight	AMRAP^†^ 2 min	Number of repetitions
Kettle-bell Swings	W: 16 kg—M: 24 kg	AMRAP^†^ 3 min	Number of repetitions
Burpees	Body Weight	AMRAP^†^ 3 min	Number of repetitions
Workout # 2 (Strength-Based).			
Movement	Load	Type	
Front Squat	Maximal	5RM	Maximal weight
350-meter Row*	—	AMRAP^†^ 15 min	Combined total number of repetitions in allotted time.
15 Burpees	Body Weight		
Workout # 3 (Metabolic Conditioning)			
Movement	Load	Type	Fastest time to completion
Deadlift	F: 61 kg M: 93 kg	21/15/9/6/3 15-min time cap	
Wall balls‡	F: 6 kg; 9 ft M: 9 kg; 10 ft		
Sit-ups	Body Weight		

* 10 meters = 1 repetition

† AMRAP = As Many Repetitions as Possible

‡ 9-feet/10-foot target for female & males

^ All scores were converted into a rate (repetitions per minute) for comparisons.

### High-Intensity Functional Training intervention

In total, 205 workouts were prescribed during the 16-week training intervention. It was typical for most training sessions to include two workouts: a strength workout [105 workouts (51%)] followed by a metabolic conditioning workout [100 workouts (49%)]. On occasion, only one of these workout-styles would be prescribed. The strength portion typically involved core, multi-joint power (e.g., squats and deadlifts) or Olympic (e.g., cleans and snatches) lifts prescribed at a variety of intensity loads (range: 70–100% of 1-RM) and volumes (range: 1–8 repetitions per set). The metabolic conditioning segments also employed core, multi-joint power and Olympic lifts, but also included a combination of cardiovascular (mono-structural), skill-based (e.g., double-unders and rope climbing), and body weight (e.g., push-ups and pull-ups) exercises. Overall, 46% of workouts were multimodal in nature, and 39% and 14% of all the workouts included either an upper body or lower body modality (See S2 Appendix).

These workouts were each designed with either a single-element (modality), a task, or a time priority to simultaneously provide metabolic, strength and neuromuscular stimulus [31]. Briefly, single element workouts will either require maximal or near maximal efforts with sufficient recovery times, focus on a specific skill (e.g. rope climbs), or long, distance efforts (e.g. row 2000 meters). Task priority workouts present a challenge (e.g., complete a 21-15-9 repetition scheme in the shortest time possible) and leave it up to the trainee to determine their strategy (i.e., pace) to accomplish the task. Time priority workouts provide the athlete a set of movements and they have to complete as many repetitions as possible (i.e., AMRAP) of each movement within the specified time period. These workouts may be designed in such a way that allows the individual to cycle through each movement at a pace that is consistent with their functional capacity. The training facility designed the workouts to build general fitness, following a general physical preparedness model and utilizing multi-joint exercises that involved the greatest number of muscle groups. Further, because individual ability (e.g., strength or the ability to perform specific exercises) among participants varied, all prescribed movements had a scaled option (i.e., the training facility’s instructor modified movements when necessary so that each participant, regardless of skill, was able to complete all prescribed repetitions). An overview of the exercises and their programming frequency during the 16-wk training intervention are presented in Table 2.

**Table 2:** Total number of workouts (and percentages) for the 16-week intervention.

Cardiovascular	n*	%^	Body Weight	n*	%^
Running	51	24.9%	Chin-up/Pull-up	71	34.6%
Jumping Rope	29	14.2%	Burpees	32	15.6%
Rowing	27	13.2%	Push-ups	24	11.7%
			Box Jumps	22	10.7%
Resistance	n*	%^	Sit-ups	21	10.2%
Squat	64	31.2%	Medicine Ball Work	20	9.8%
Clean	33	16.1%	Handstand/HRPU	18	8.8%
Snatch	33	16.1%	T2B	16	7.8%
Deadlift	29	14.2%	Rings	8	3.9%
KB Swings	24	11.7%	Rope Work	8	3.9%
Thrusters	21	10.2%	TGU	3	1.5%
Lunges	19	9.3%	Muscle Ups	2	1.0%
Bench Press	17	8.3%			
Loaded Row	8	3.9%	Auxiliary work	n*	%^
Stability Row	8	3.9%	Back Extensions	5	2.4%
Jerk	8	3.9%	Floor Sweeps	5	2.4%
Press	6	2.9%	Overhead Sledge Strikes	5	2.4%
Curls	5	2.4%	Russian Twists	3	1.5%
Farmer Walks	5	2.4%	Bar Roll-outs	2	1.0%
Romanian Dead Lift	2	1.0%	Prowler Push	2	1.0%
Triceps Extensions	2	1.0%	^†^Other	1	0.5%

KB = kettle bell; HRPU = hand release push-ups; T2B = toes to bar; Turkish Get-ups

* n = the number of workouts an exercise appeared in programming

^ % = the percentage of workouts that utilized an exercise.

^†^ Includes single sessions of: Bear crawls; dumbbell man makers; Glute bridges; dumbbell lateral raises; Plank holds; wood chops with band; Kettle bell Cross walks

## Statistical analysis

To assess the effect of the training intervention and sex, separate two-way (Sex x Time) repeated measures analyses of variance (RMANOVA) were performed on all total body measures of composition and measures of sports-specific performance. To further assess the effect of sex and the intervention on regional composition, separate three-way RMANOVA’s (Region x Time x Sex) were performed where region consisted of 3 levels (i.e., ARM, LEG, TRNK) for BMC and BMD, and 2 levels (i.e., ARM and LEG) for lean mass; Sex (i.e., male or female) and Time (i.e., PRE and POST) each consisted of 2 levels. Where violations of the sphericity assumption occurred, the Greenhouse-Geisser procedure was used to adjust degrees of freedom. Significant main effects, interactions, and post-hoc analyses were assessed using Bonferonni adjustments. All between group differences were further analyzed using effect sizes (η^2^: Partial eta squared). Interpretations of effect size were evaluated [32] at the following levels: small effect (0.01–0.058), medium effect (0.059–0.137) and large effect (> 0.138). A criterion alpha level of p ≤ 0.05 was used to determine statistical significance. All data are provided in S1 Appendix and reported here as mean ± standard deviation (M ± SD). Statistical Software (V. 22.0, SPSS Inc., Chicago, IL) was used for all analyses.

To examine if the changes for all regional measurements (i.e., ARM, LEG, TRNK) could be considered real, all individual change scores (for BMD, BMC, and NBLM) were compared to their calculated minimal difference (MD) [33]. Using the equation for MD (MD = Standard Error of the Measurement x 1.96 x √2) we created 95% confidence interval about the standard error of the measurement (SEM). Any change occurring within this confidence interval would be interpreted as being consistent with the measurement error of the test, while changes occurring outside of the interval reflect real changes in body composition.

## Results

Of the fifty-three original participants who volunteered for the study, a total of nine males (34.2 ± 9.1 y; 91.5 ± 17.7 kg; 178.5 ± 5.4 cm) and 17 females (36.4 ± 7.9 y; 91.5 ± 17.7 kg; 162.9 ± 7.0 cm) completed all testing measures after the 16-weeks of training. The attrition rate (49%) reported in this study is similar to that reported by other investigators [34]. However, this attrition was calculated due to “nonattendance” to post testing measurements, not necessarily failure to complete the 16-weeks of training. In addition, no major injuries were reported by any of the participations during the training period.

Overall, our participants were recreationally trained individuals with over 16 months of HIFT training experience (16.38 ± 14.02 months). Although not statistically different (F = 0.469, p = 0.50, η^2^ = 0.019), males reported longer training experience than females (19.0 ± 18.2 vs. 15.0 ± 11.7 months). Most participants attended the fitness facility 3–5 days per week throughout this study.

### Total body composition measures

Changes in total body composition are presented in Table 3. A significant (time x sex) interaction (F = 5.6, p = 0.027, ɳ^2^_p_ = 0.19) was observed for BMC, where improvements favored women (1.0 ± 1.1%, p = 0.004) over men (-0.1 + 0.8%, p = 0.625). While no other significant (time x sex) interactions were observed for any of the body composition measures, a significant main time effect (F = 4.3, p = 0.048, ɳ^2^_p_ = 0.15) was observed for %FAT. Following training, a 4.6 ± 12.4% reduction in %FAT was observed in all participants without any significant changes to body mass (–0.6% ± 3.7%, p = 0.450) or NBLM (0.5 ± 4.0%, p = 0.778). Rather, a trend (F = 3.2, p = 0.088, ɳ^2^_p_ = 0.12) was noted for reductions in total fat mass (–4.6 ± 16.2%). Additionally, a trend (F = 3.6, p = 0.072, ɳ^2^_p_ = 0.13) was noted for improvements to occur in BMD for all participants (PRE: 1.25 ± 0.13 g · cm^2^; POST: 1.26 ± 0.13 g · cm^2^).

**Table 3:** Changes in total body composition following 16-wks of high intensity functional training.

	PRE	POST	%Change	F	p-value	ɳ^2^_p_
*Body Fat Percentage (%)*						
Females (n = 17)	31.29 ± 7.29	30.08 ± 8.33	-6.1 ± 13.2	0.094	0.762	0.004
Males (n = 9)	24.49 ± 7.49	22.87 ± 6.63	-7.5 ± 16.8			
Total (n = 26)	28.93 ± 7.93	27.58 ± 8.41	-6.5 ± 14.2	4.338	0.048	0.153
*Fat Mass (kg)*						
Females (n = 17)	21.18 ± 8.17	20.44 ± 9.34	-7.2 ± 18.5	0.569	0.458	0.023
Males (n = 9)	22.44 ± 11.51	20.61 ± 10.26	-8.9 ± 19.8			
Total (n = 26)	21.62 ± 9.24	20.5 ± 9.46	-7.8 ± 18.6	3.157	0.088	0.116
*Bone-free lean mass (kg)*						
Females (n = 17)	44.63 ± 6.59	45.29 ± 6.86	1.4 ± 2.2	2.232	0.148	0.085
Males (n = 9)	66.75 ± 7.57	65.78 ± 7.25	-1.6 ± 6.6			
Total (n = 26)	52.29 ± 12.7	52.38 ± 12.07	0.3 ± 4.4	0.081	0.778	0.003
*Bone mineral density (g · cm^-2^)*						
Females (n = 17)	1.19 ± 0.08	1.19 ± 0.08	0.7 ± 1.9	1.469	0.237	0.058
Males (n = 9)	1.36 ± 0.13	1.4 ± 0.11	2.7 ± 7.0			
Total (n = 26)	1.25 ± 0.13	1.26 ± 0.13	1.4 ± 4.4	3.552	0.072	0.129
*Bone mineral content (g)*						
Females (n = 17)	2485 ± 320	2508 ± 322	0.9 ± 1.1	5.558	0.027	0.188
Males (n = 9)	3556 ± 342	3551 ± 346	-0.2 ± 0.8			
Total (n = 26)	2856 ± 611	2869 ± 600	0.6 ± 1.1	2.324	0.140	0.088

Note: Inferential statistics for assessing the (sex x time) interaction and the main effect for time are separated into the upper and lower sections, respectively.

### Regional body composition measures

A significant (region x time x sex) interaction (F = 4.0, p = 0.026, ɳ^2^_p_ = 0.15) was observed for BMC but not BMD (F = 0.7, p = 0.457, ɳ^2^_p_ = 0.03) or NBLM (F = 0.1, p = 0.763, ɳ^2^_p_ < 0.01). Although BMC was significantly (p < 0.001) greater in men compared to women at all PRE- and POST-regional locations (i.e., ARM, LEG, and TRNK), greater improvements in BMC_TRNK_ were found in women (2.5 ± 3.0%, p < 0.005) but not men (-0.3 ± 1.8%, p = 0.621). Changes in BMC_LEG_ were comparable between women (0.8 ± 1.0%, p < 0.001) and men (0.3 ± 0.6%, p < 0.001), while no changes were observed in BMC_ARM_. However, it is noteworthy that none of the observed changes in BMC measures exceeded their respective MD score. All changes in regional composition measures are presented in Table 4.

**Table 4:** Regional changes in bone-free lean mass and bone mineral characteristics following 16-wks of high intensity functional training.

		PRE	POST	Change	MD	%Exceeding MD
*Bone-free lean mass (kg)*						
ARMS	Females (n = 17)	4.70 ± 0.80	4.90 ± 0.91	0.20 ± 0.30	0.43	11.8
	Males (n = 9)	8.57 ± 0.81	8.49 ± 0.79	-0.09 ± 0.25		0.0
	Total (n = 26)	5.94 ± 2.01	6.05 ± 1.91	0.11 ± 0.31		8.0
LEGS	Females (n = 17)	18.68 ± 3.15	18.96 ± 3.53	0.28 ± 0.73	1.43	5.9
	Males (n = 9)	26.87 ± 3.06	26.77 ± 3.14	-0.11 ± 0.87		0.0
	Total (n = 26)	21.52 ± 5.01	21.66 ± 5.05	0.14 ± 0.79		3.8
*Bone mineral density (g · cm^-2^)*						
ARMS	Females (n = 17)	0.73 ± 0.06	0.80 ± 0.26	0.07 ± 0.25	0.09	6.3
	Males (n = 9)	0.92 ± 0.07	0.92 ± 0.10	0.01 ± 0.10		12.5
	Total (n = 26)	0.79 ± 0.11	0.84 ± 0.22	0.05 ± 0.21		8.3
LEGS	Females (n = 17)	1.25 ± 0.09	1.25 ± 0.09	0.01 ± 0.03	0.14	0.0
	Males (n = 9)	1.51 ± 0.08	1.53 ± 0.09	0.02 ± 0.02		0.0
	Total (n = 26)	1.34 ± 0.15	1.35 ± 0.16	0.01 ± 0.02		0.0
TRNK	Females (n = 17)	1.13 ± 0.11	1.15 ± 0.11	0.02 ± 0.03	0.05	18.8
	Males (n = 9)	1.32 ± 0.12	1.27 ± 0.26	-0.05 ± 0.18		25.0
	Total (n = 26)	1.20 ± 0.14	1.19 ± 0.18	-0.01 ± 0.11		20.8
*Bone mineral content (g)*						
ARMS	Females (n = 17)	299 ± 43	299 ± 43	0.2 ± 6.4	46.8	0.0
	Males (n = 9)	484 ± 45*	485 ± 47*	1.1 ± 5.5		0.0
	Total (n = 26)	361 ± 99	361 ± 99	0.5 ± 6		0.0
LEGS	Females (n = 17)	904 ± 146	911 ± 145#	6.5 ± 8.3	26.9	0.0
	Males (n = 9)	1362 ± 138*	1366 ± 136*#	3.9 ± 8.9		0.0
	Total (n = 26)	1063 ± 263	1068 ± 261	5.6 ± 8.4		0.0
TRNK	Females (n = 17)	509 ± 84	522 ± 87#	12.4 ± 15	40.2	0.0
	Males (n = 9)	723 ± 90*	720 ± 87*	-2.4 ± 13		0.0
	Total (n = 26)	580 ± 133	588 ± 128	7.5 ± 15.8		0.0

Note: ARMS = both arms; LEGS = both legs; TRNK = spine and pelvis; MD = Minimal difference

* = Significantly (p < 0.05) different from females

# = Significantly (p < 0.05) different from PRE.

### Performance measures

**Fig 1:**
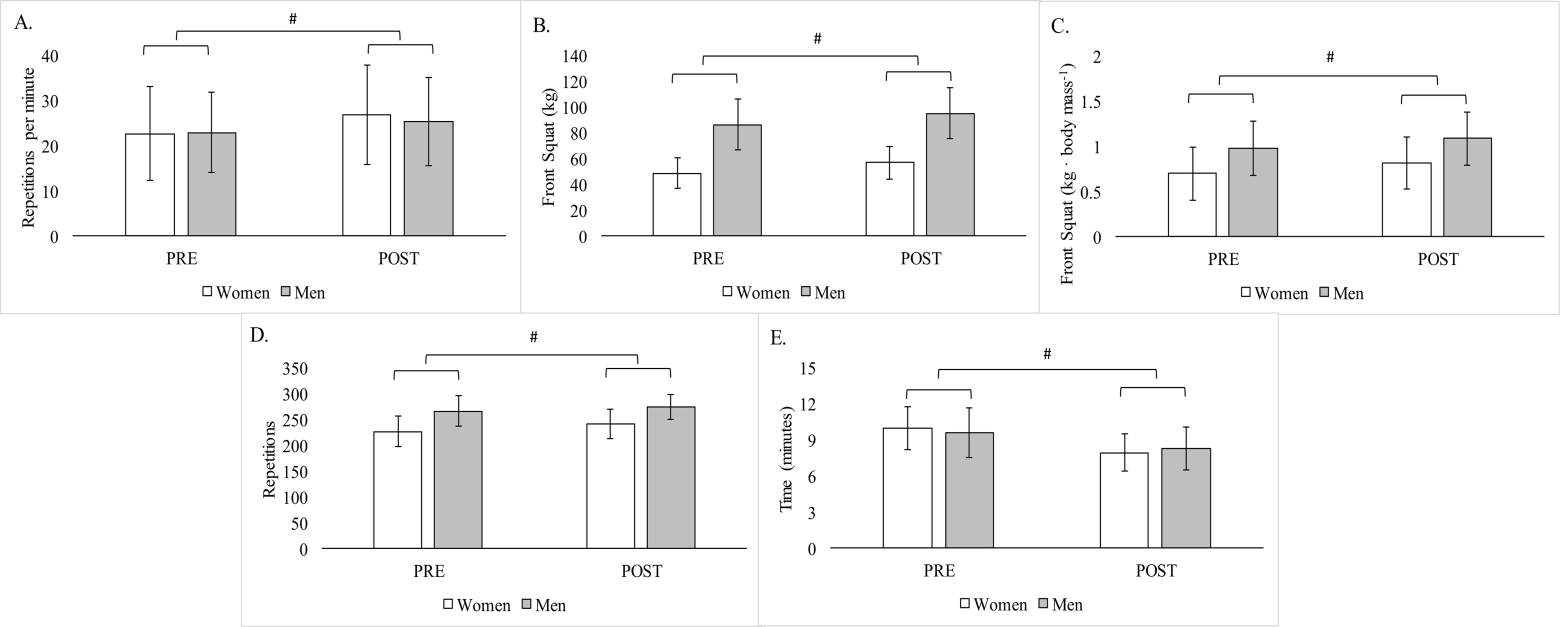
Changes in performance during. (A) WOD-1, (B) Absolute 5-RM Front Squat, (C) Relative 5-RM Front Squat, (D) WOD-2, and (E) WOD-3 following 16-wks of high-intensity functional training. Note: # = Significantly (p < 0.05) different from PRE.

No (time x sex) interactions were noted for any of the performance measures (Fig 1). However, significant main effects for time (p < 0.001) were found in each performance measures when the data were collapsed across groups. Following the 16-weeks of HIFT, improvements were observed in the average repetition rate during WOD-1 (18.3 ± 16.8%), 5RM front squat strength (absolute: 14.4 ± 9.7%; relative: 15.4 ± 9.2%), the total number of repetitions completed during WOD-2 (5.7% ± 6.5%), and a reduced time to completion during WOD-3 (–17.3% ± 14.7%).

## Discussion

The present study investigated the effect of HIFT for 16-weeks on body composition, bone formation, and sport-specific performance. Only a pair of studies had previously documented improvements in body composition and performance following 5- [9] and 12 weeks [10] of HIFT. In part, our findings are consistent with these studies. Here, we observed improvements in performance and overall body composition via reduction in body fat mass following 16-wks of training. However, we did not observe uniform changes in lean and skeletal mass across all body regions. To the best of our knowledge, our study appears to be the longest to examine HIFT outcomes and the first monitor changes in bone mass.

The observed changes in body fat percentage and trends towards reduced fat mass, without significant changes in total body mass would suggest improvements in lean mass also occurred; however, changes in lean mass were not observed. In fact, only 8.0% (or 3 out of 26) and 3.8% (or 1 out of 26) participants experienced a change that exceeded the minimal difference for lean arm mass or lean leg mass, respectively. These findings are inconsistent with previous reports of improvements in lean mass among cancer survivors [9] and healthy individuals [10]. It is possible that differences between study populations (i.e., healthy adults versus a clinical population) [9] and variability in training status and ability (i.e., historical and current experience) among recreationally-active, healthy adults may explain these differences. Moreover, our study participants had been exposed to this type of training an average of 16-months, which would suggest most were under a “maintenance” stage, and not necessarily trying to modify body composition [35].

It is also worth pointing out that since our participants were able to scale each workout to their individual ability, the exact intensity and complexity employed by each participant may have also varied. Unfortunately, we were unable to provide an exact dose of training for each participant during the 16-weeks, but we would expect that those individuals with more experience may have required less modifications during each workout, and thus, would have remained more consistent with the specific goals of each workout. Future studies performed in a similar setting should consider this limitation and make every effort to document each participants workout in order to provide an accurate representation of the actual dose of training performed throughout the study. Additionally, daily protein and caloric intake are known to influence changes in body composition and lean mass [36]. Although these were not monitored or controlled in the present investigation, significant dietary changes were not expected as participants had already been consistently training for more than a year. Nevertheless, nutritional and caloric intake assessment warrants inclusion in future investigations.

Interestingly, changes in skeletal mass did not mirror those in NBLM. Following 16-weeks of HIFT, adaptations were primarily observed in the lower limb (men and women) and trunk (women only). Previously, Conroy et al. (8) reported greater bone mineral density values of the lumbar spine and proximal femur in elite junior Olympic weightlifters compared to controls and reference data. Considering that HIFT programming typically employs Olympic lifting and that the primary aim is to elicit widespread adaptations [24], skeletal improvements in all regions were expected. Instead, adaptations were primarily observed in the trunk and lower limb, which was likely the result of the specific programming. During the 16-wk period, exercises that loaded the spine and lower body (e.g., squats, deadlifts, cleans) occurred approximately 14.2–31.2% of the time. Though upper-body movements also occurred in high frequency (11.7–34.6% of classes), these were typically body weight or gymnastic-type movements (e.g., pull-ups, muscle-ups, push-ups). In fact, the most frequently-used load bearing exercises that stressed the upper limb (i.e., thrusters and bench press) only occurred on 10.2% and 8.3% of classes, respectively. It is possible, however, that a longer training duration would have elicited more comprehensive improvements. HIFT closely resembles a non-linear periodization design, where programming attempts to elicit adaptations across a wide variety of physiological outcomes. Consequently, the trainee might complete several workouts in succession that do not meet the needs of a specific training outcome (e.g., upper body skeletal mass improvement). Comparatively, a linear periodization model focuses on more specific adaptations and thus, appropriate programming occurs more frequently. While the superiority of either training model is unclear, it is generally thought that non-linear designs require longer durations to observe improvements [37].

The duration of the study may also have been insufficient to observe meaningful changes to skeletal structure due to the time requirements for skeletal remodeling. Robling and colleagues [38] reported structural changes that supported increased bone strength following 16-weeks of loading in rats, however, only small increases in BMC and BMD were observed. The ACSM indicates that a minimum of 6–8 months of consistent training is necessary to detect a new steady-state bone mass [39]. However, even this length of time does not guarantee that an osteogenic effect has occurred. For instance, despite having approximately 2.5 years of resistance training experience, Tsuzuku et al. [40] found that BMD in 10 collegiate power lifters only differed in the lumber spine compared to 11 sedentary controls. It is also possible that the limitations of the DXA to detect adaptations may have contributed to our observed outcomes. For instance, Fujimar and colleagues [41] reported changes in bone metabolism biomarkers (i.e., osteocalcin, bone-specific alkaline phosphatase) following a similar training duration, but were not able to detect adaptations via DXA scan. In the present investigation, though significant changes were noted, the observed changes did not exceed the minimal difference necessary to exceed measurement error. Thus, further investigation using a longer training duration appears warranted.

In addition to stimulating a wide variety of physiological improvements, HIFT aims to improve performance over a broad spectrum of physical demands. Following 16 weeks of HIFT, participants in the present study increased absolute and relative 5RM front squat strength and performance in all workout challenges. These findings are in agreement with those reported by Heinrich and colleagues [11] who compared a HIFT program to traditional military training protocol in military personal and reported greater improvements during a 2-minute push-up test (4.2 ± 5.4 vs 1.3 ± 5.9), 2-mile run (-89.91 ± 70.23 vs -15.33 ± 69.16 seconds), 1RM bench press (13.2 ± 12.1 vs 2.7 ± 11.5 pounds), and flexibility (seat and reach; 0.6 ± 1.3 vs -0.5 ± 1.5 inches) following HIFT. Likewise, Buckley et al. [8] observed greater improvements following a multimodal high-intensity interval training protocol (MM-HIIT) compared to a row high-intensity training protocol (Row-HIIT) in recreationally-active females in muscle power (broad jump; 6%), 1RM strength (back squat; 39%, overhead press; 27%, and deadlift; 18%), and muscle endurance (back squat repetitions to failure at 70% 1RM; 280%). While it is possible that the observed performance changes were the consequence of specific adaptations to the imposed demands of training, these were likely to have been negated by training experience. HIFT protocols typically vary across training facilities and research investigations. However, their design and exercise composition are generally consistent. Thus, individuals with experience should have been relatively familiar with the specific demands of performance tests at baseline, as well as any potential strategies they might use to maximize performance.

To our knowledge, this is the first study to present longitudinal data reporting changes in body composition and performance after 16-weeks of HIFT–most other studies only report 4 to 12 weeks. Overall, our findings support the notion that HIFT is an efficient and effective strategy for simulating adaptations across a variety of physiological and performance measures. These findings may be of interest to athletes, coaches, and other fitness industry enthusiasts and professionals who are looking to elicit several adaptations without being confined by the rigid structure and time commitment of the traditional linear periodization model. Considering the growth of HIFT models over the last decade, it is important to continue to examine the role of this training modality in specific health outcomes.

## Supporting information

S1 Appendix (SAV)

S2 Appendix (PDF)
