# Supplementary material for: Changes in body composition, bone metabolism, strength, and skill-specific performance resulting from 16-weeks of HIFT
Source: PLoS One. 2018 Jun 15;13(6):e0198324. doi: 10.1371/journal.pone.0198324 (PMC6003684; doi:10.1371/journal.pone.0198324)
Supplement: S2 Appendix — (PDF) [file pone.0198324.s002.pdf]

	Weights (lbs)	Time (min)	Reps	Distance	Sets		Sums	NOTES
105								
100								
Day 1								
Strength:								
Single Arm DB Press			8each		4		1	Press (DB, Single)
DB Bench Row			8each		4		1	Bench Row (DB)
MetCon:								4 Rounds
Run 400 m				400m	4		45	Run
Power Clean (135/85, 95/65) x 10	(135/85, 95/65)		10		4		16	Clean (Power)
Sit-ups x 20			20		4		16	Sit-ups
Day 2								
Testing:								
Thrusters (115/75) x 20 reps	(115/75)	4	20				20	Thrusters
Rest x 2 mins		2						
Max effort DU's x 2 mins		2			1		26	DU's
Rest 2 mins		2						
Max effort KB Swings (24/16) x 2 mins	(24/16)	2			1		19	KB Swings
Rest x 2 mins		2						
Max effort Burpees to 45lb. Plate x 3 mins	45lb. Plate	3			1		3	Burpees (To Plate)
Day 3								
Strength:								
Snatch balance & OHS 1 by 3 x 5			1		5		14	Snatch
OHS 3 x 5			3		5		6	Squat (Overhead)
MetCon:								
3 rounds								
Pull-ups x 15			15		3		22	Pull-ups
HSPU x 10			10		3		15	HSPU
DL (295/200, 245/175) x 5	(295/200, 245/175)		5		3		27	Deadlift
Day 4								
Strength:								
Front Squat x 5RM	5 RM	20					17	Squat (Front)
MetCon: 15 min AMRAP								
Row 350 m		15		350m	AMRAP		26	Row
Burpees x 15		15	15		AMRAP		23	Burpees
Day 5								
Strength: EMOM								
Power Clean & Jerk x 3 @ 60-70%	(60-70%)	7	3				2	Clean (Power) and Jerk
MetCon: 4 Rounds								
Ring Chin-ups x 8			8		4		1	Chin-ups (Ring)
OH Walking DB Lunge x 10	(35/25, 30/20)		10		4		4	Lunges (OH Walking)
Day 6								
Strength:								
DL x 2/set	60%		2		1			Deadlift
	65%		2		1			
	70%		2		1			
	75%		2		1			
	80%		2		1			
MetCon: 21-15-9-6-3								
DL (205/135)	(205/135)		21-15-9-6-3					Deadlift
WB (20/14)	(20/14)		21-15-9-6-3				15	Wall Balls
Sit-ups			21-15-9-6-3					Sit-ups
Day 7								
Strength:								
Squat	(70-75%)		5		5		18	Squat
MetCon:								
	(25/15)			200m	1		3	Run (Plate)
Squat Cleans	(95/65)		30		1		11	Squat Clean
Plate Run	(45/25)			200m	1			Run (Plate)
Squat Cleans	(135/85)		20		1			Squat Clean
Plate Run	(25/15)			200m	1			Run (Plate)
Squat Cleans	(185/115)		10		1			Squat Clean
Push-ups			50		1		15	Push-ups
DU's			100		1			DU's
T2B			40		1		15	T2B
DU's			80		1			DU's
Box Jumps			30		1		17	Box Jumps
DU's			60		1			DU's
Day 8								
Strength:								
BB Walking Lunge	AHAP			20paces	3		3	Lunges (BB Walking)
BB Walking Lunge	AHAP			10paces	2			Lunges (BB Walking)
BB Walking Lunge	AHAP			10paces	2			Lunges (BB Walking)
MetCon:								
Double Bell KB Jerk	(32/20, 24/16)		15		4		2	Jerk (Double Bell KB)
Single Ring, Ring Rows			15		4		6	Ring Rows (single)
KB Farmer Walk				100m	4		2	Farmer Walks (KB)

	Weights (lbs)	Time (min)	Reps	Distance	Sets		Sums	NOTES
Day 9								
Strength:								
Bench Press	50%		5		1		14	Bench Press
	60%		5		1			
	70%		5		1			
	85%		5		1			
	100%		3		1			
	70%		8		1			
MetCon:								
WB			Max					Wall Balls
Rest		3						
KB Swings	(32/24, 24/20)		10		5			KB Swings
KB Goblet Squats			10		5		3	Squat (KB Goblet)
Pull-ups			10		5			Pull-ups
Day 10								
Strength:								
Squat Clean	?		1		5			Squat Clean
Front Squat	?		2		5			Squat (Front)
Jerk	?		1		5		4	Jerk
MetCon:								
HSPU			8		4			HSPU
Box Jumps			8		4			Box Jumps
Push-ups			8		4			Push-ups
DU's			25		4			DU's
Day 11								
Strength:								
Back Squat	50%		12		1			Squat
	increase 10-20lbs.		10		1			
	increase 10-20lbs.		8		1			
	increase 10-20lbs.		6		1			
	increase 10-20lbs.		4		1			
	increase 10-20lbs.		2		1			
MetCon:								
Run		20		400m	AMRAP			Run
Burpees to 45/25lb. Plate	45/25lb. Plate	20	5		AMRAP			Burpees (To Plate)
Walking Lunge		20	16		AMRAP		1	Lunges (Walking)
Burpees to Plate	45/25lb. Plate	20	5		AMRAP			Burpees (To Plate)
Day 12								
Strength:								
3 Position Snatch	50%		1		3		1	Snatch (3 Position)
Hang Snatch	?		2		3		5	Snatch (Hang)
Snatch	?		1		3			Snatch
MetCon:								
Power Cleans	(185/25, 135/85)		21, 15, 9		3			Clean (Power)
S2OH			21, 15, 9		3			Shoulder 2 Over-Head (S2OH)
Day 13								
Strength:								
Sumo DL	60%		5		1		2	Deadlift (Sumo)
	70%		4		1			
	75-85%		3		3			
MetCon:								
Ladder		12Min.						
Strict Chin-ups							12	Chin-ups (Strict)
HRPU			2,4,6,8,10...				8	HRPU
Sit-Ups			4,8,12,16,20...					Sit-ups
Day 14								
Strength:								
Front Squat:	60%		5		1			Squat (Front)
	70%		5		1			
	75%		5		1			
	80%		5		1			
	85%		3		1			
	75%		8		1			
MetCon:								
Power Snatch	(115/75, 95/65)		10		5		5	Snatch (Power)
Thrusters			20		5			Thrusters
KB Swings	(32/24, 24/20)		30		5			KB Swings
Run				200m	5			Run
Day 15								
Strength:								
Loaded Ring Rows	loaded		8 to 10				2	Ring Rows
Strict T2B			5 to 8				1	T2B (Strict)
MetCon:								
Handstand Holds		30seconds			4			Handstand Holds
Bear Crawls				D&B	4		1	Bear Crawls
DB Step-ups	(35/25)		10		4		2	Step-ups (DB)
Running Man Jump Rope			50		4			Running Man Jump Rope
Day 16								
Strength:								

	Weights (lbs)	Time (min)	Reps	Distance	Sets		Sums	NOTES
Bench Press	50%		5		1			Bench Press
	65%		5		1			
	75%		5		1			
	90%		5		1			
	100%		5		1			
MetCon:								
HSPU		3	max reps					HSPU
Scale-HRPU?								HRPU
Rest			3min					
Bar Muscle Ups		9	5		AMRAP		2	Muscle Ups
Power Cleans	(185/125, 135/95)	9	7		AMRAP			Clean (Power)
			9		AMRAP			Burpees
Burpee Box Jumps	(30/24, 24/20)	9	9		AMRAP			Box Jumps
Day 17								
Strength:								
Snatch	(75-85%)		1		10			Snatch
MetCon:								
KB Swings	(32/24, 24/20)		20		4			KB Swings
Goblet Squat			15		4			Squat (KB Goblet)
OH Single Arm Walking Lunge	Single Arm			10paces	4		2	Lunges (OH Walking, Single)
Day 18								
Strength:								
Front Squat	50%		5		1			Squat (Front)
	60%		5		1			
	70%		5		1			
	85%		5		1			
	95%		5		1			
MetCon:								
Row				1000m	1			Row
Wall Balls			25		3			Wall Balls
DL	(225/155, 185/125)		15		3			Deadlift
T2B			10		3			T2B
Day 19								
Strength:								
Power Cleans	(185/125, 135/95)	12	5					Clean (Power)
Bar Over Burpees	Bar Over	12	5				1	Burpees (Bar Over)
MetCon:								
S2OH	(185/125, 135/95)		5		5			Shoulder 2 Over-Head (S2OH)
Ring Dips			7		5		7	Ring Dips
DU's			35		5			DU's
Day 20								
Strength:								
DL	50%		5		1			Deadlift
	60%		4		1			
	70%		3		5			
MetCon:								
Power Snatch	(95/65, 75/55)		15		3			Snatch (Power)
Single Ring-Ring Rows			15		3			Ring Rows (single)
Sit-ups w/DB	(35/25, 30/20)		15		3		1	Sit-ups (DB)
Day 21								
Strength:								
Back Squat	50%		10		1			Squat
	60%		8		1			
	70%		6		4			
MetCon:								
Pull-ups			25		1			Pull-ups
DL	(135/95)		50		1			Deadlift
Push-ups			50		1			Push-ups
Box Jumps			50		1			Box Jumps
Floor Sweeps			50		1		5	Floor Sweeps
KB Clean and Press	(24/16)		50		1		1	Clean (KB) and Press
Pull-ups			25		1			Pull-ups
Day 22								
Strength:								
Single Arm DB/KB OHS	?		8each		3			Squat (Overhead, single, DB/KB)
KB TGU (AHAP)	(AHAP)		2each		3			TGU (KB)
MetCon:								
Run		20		200m				Run
Strict Chin-ups		20	5					Chin-ups (Strict)
Run		20		400m				Run
DB 1/2 Man maker	(35/25, 30/20)	20	5					DB 1/2 Man Maker
Day 23								
Strength:								
Bench Press	50%		5		1			Bench Press
	65%		5		1			
	80%		5		1			
	95%		5		1			
	105%		3		1			
	80%		8		1			
MetCon:		10min cap						
HSPU			10		4			HSPU

	Weights (lbs)	Time (min)	Reps	Distance	Sets		Sums	NOTES
DL	?		10		4			Deadlift
Day 24								
Strength:								
Hang Clean			1		6		2	Clean (Hang)
Clean			1		6		2	Clean
Jerk			1		6			Jerk
MetCon:								
Wall Balls	?		21-15-9-15-21					Wall Balls
KB Swings	?		21-15-9-15-21					KB Swings
Unbroken DU's	Unbroken		25					DU's
Day 25								
Strength:								
Front Squat	60%		5		1			Squat (Front)
	70%		5		1			
	80%		5		1			
	90%		5		1			
	100%		3		1			
	80%		8		1			
MetCon:								
Pull-ups			10		3			Pull-ups
Box Jumps			10		3			Box Jumps
T2B			10		3			T2B
			10		3			
Burpee Box Jumps			10		3		2	Burpee Box Jumps
Day 26								
Strength:								
Pause Snatch (Pos. 2)	Pause @ pos. 2		2		6			Snatch
MetCon:								
Row				1500m	1			Row
KB Double Bell Squat (32/20, 24/16)	(32/20, 24/16)		30		1			Squat (KB/DB)
Push-ups	45lb		30		1			Push-ups
KB double bell lunge	?		30		1			Lunges (KB/DB)
Day 27								
Strength:								
Clean Pulls			3		6		1	Clean (Pull)
MetCon:								
Run				400m	4			Run
Thrusters	(135/85, 95/65)		10		4			Thrusters
Sit-ups			20		4			Sit-ups
Day 28								
Strength:								
Rope Climbs		20			1		7	Rope Climbs
Rx			15accents		1			
FB (legless + traditional)			5 + 10		1			FB (legless + traditional)
MetCon:								
DB Bench Press	(35/25, 30/20)		40		1		3	Bench Press (DB)
Squats	40%		30		1			Squat
Strict Chin-ups			20		1			Chin-ups (Strict)
DB Bench Press			20		1			Bench Press (DB)
Squats	60%		15		1			Squat
Strict Chin-ups			10		1			Chin-ups (Strict)
Day 29								
Strength:		(10min EMOM)						
Pistols			5each		Odd Minute			Squat (Pistol)
Strict HSPU			5		Even Minute		2	HSPU (Strict)
MetCon:								
Run			800m		1			Run
Double Bell KB Swings (32/20, 24/16)	double bell (32/20, 24/16)		10		4			KB Swings
Burpees			10		4			Burpees
Run			800m		1			Run
Day 30								
NO W.O.D. - Holiday								
Day 31								
Strength:								
Squat Clean + Thruster			1		6			Squat Clean
			3		6			Thrusters
MetCon:								
S2OH	(135/85, 95/65)		15-12-9					Shoulder 2 Over-Head (S2OH)
Pull-ups			15-12-9					Pull-ups
S2OH			12,9,6					Shoulder 2 Over-Head (S2OH)
Pull-ups			12,9,6					Pull-ups
S2OH			9,6,3					Shoulder 2 Over-Head (S2OH)
Pull-ups			9,6,3					Pull-ups
Day 32								
Strength:								
Back Squat @ 80%	@80%		2		6			Squat

	Weights (lbs)	Time (min)	Reps	Distance	Sets		Sums	NOTES
MetCon:		(For Time)						
DU's			100		1			DU's
Row			1000m		1			Row
KB Swings (32/20, 24/16)	(32/20, 24/16)		100		1			KB Swings
Air Squats			100		1		3	Squat (Air)
Day 33								
Strength:								
Snatch Pulls + Snatch			2		5		2	Snatch Pull
			2					Snatch
MetCon:								
Hang Power Cleans (155/105, 115/85)	(155/105, 115/85)		10		3		4	Clean (Hang Power)
BB OH Walking Lunges (155/105, 115/85)	(155/105, 115/85)		10		3			Lunges (OH Walking)
Lateral Burpees over bar	Bar Over		10		3		2	Burpees (Lateral Bar Over)
Day 34								
Strength:		(6min EMOM)						
3 DL (Increase 5% ea. Min.)	(Increase 5% ea. Min.)		3		6			Deadlift
MetCon:								
Squat Cleans (205/135, 155/95)	(205/135, 155/95)		3		5			Squat Clean
HSPU			6		5			HSPU
Bowtie situps (35/25, 30/20)	(35/25, 30/20)		9		5		3	Sit-ups (Bowtie)
Day 35								
Strength:								
Front Squat								
60%			5		1			Squat (Front)
65%			5		1			
70%			5		1			
75%			5		1			
80%			5		1			
70%			8		1			
MetCon:								
Run			200m		3			Run
Slam Balls			20		3		5	Ball Slams
No Rest								
Run			400m		3			Run
Strict Chin-ups			8		3			Chin-ups (Strict)
Push-ups			12		3			Push-ups
Day 36								
Strength:		(15min. EMOM)						
DB or KB SLRDL			5each		Min.1 (5)			RDL (DB/KB, single)
Single arm DB or KB Shoulder Press			5each		Min.2 (5)		1	Shoulder Press (DB/KB, Single)
Single Arm Bench Row			5each		Min.3 (5)		1	Bench Row (single)
MetCon:								
Row			250m		5			Row
Burpees			10		5			Burpees
Single arm KB Swings			20		5		5	KB Swings (single)
Day 37								
Strength:								
Bench Press								Bench Press
65%			5		1			
70%			5		1			
75%			5		1			
80%			5		1			
85%			5		1			
75%			8		1			
MetCon:								
Power Clean (135/85, 95/65)	(135/85, 95/65)		15		1			Clean (Power)
Thrusters			15		1			Thrusters
DL			15		1			Deadlift
Power Clean (155/105, 115/85)	(155/105, 115/85)		9		1			Clean (Power)
Thrusters			9		1			Thrusters
DL			9		1			Deadlift
Power Clean (175/125, 135/105)	(175/125, 135/105)		6		1			Clean (Power)
Thrusters			6		1			Thrusters
DL			6		1			Deadlift
Day 38								
Strength:								
								Snatch (Power)
Hang Power Snatch + Power Snatch			2		6		2	Snatch (Hang Power)
MetCon:		(15min AMRAP)						
Feet elevated Push-ups	Feet elevated		10		AMRAP			Push-ups
sit-ups			15		AMRAP			Sit-ups
			1		6			Snatch (Power)
KB Snatch (32/20, 24/16)	(32/20, 24/16)		20		AMRAP		2	Snatch (KB)
Run			200m		AMRAP			Run
Day 39								
Strength:								
Back Squat @ 75%	@ 75%		3		6			Squat

	Weights (lbs)	Time (min)	Reps	Distance	Sets		Sums	NOTES
MetCon:								
Unbroken DU's			30		4			DU's
Wall Balls (30/20, 20/14)	(30/20, 20/14)		15		4			Wall Balls
Pull-ups			10		4			Pull-ups
Day 40								
Strength:								
Jerks			3		5			Jerk
MetCon:		(8min. AMRAP)						
Row			200m		AMRAP			Row
HSPU			8		AMRAP			HSPU
DB Walking Lunges (35/25, 30/20)	(35/25, 30/20)		16paces		AMRAP			Lunges (DB Walking)
Day 41								
Strength:								
DL								Deadlift
60%			5		1			
65%			4		2			
70%			3		2			
75%			2		2			
80%			1		2			
MetCon:		(For time)						
Burpees			10		1			Burpees
T2B			20		1			T2B
Box Jumps			30		1			Box Jumps
KB Swings (32/24, 24/20)	(32/24, 24/20)		40		1			KB Swings
KB Loaded Step-up	Loaded		30		1		2	Step-ups (KB)
T2B			20		1			T2B
Burpees			10		1			Burpees
Day 42								
Strength:								
Front Squat								Squat (Front)
70%			5		1			
80%			5		1			
90%			5		1			
100%			5		1			
105%			5		1			
110%			3		1			
MetCon:								
Strict DB Shoulder Press (35/25, 30/20)	(35/25, 30/20)		5		5		1	Shoulder Press (DB, Strict)
Strict Chin-ups			7		5			Chin-ups (Strict)
HRPU			9		5			HRPU
Running man single-unders			50		5		2	Running man single-unders
Day 43								
Strength:								
Bent BB Rows			8		6		2	Bent Row (BB)
Ring Dips			5 to 8		6			Ring Dips
MetCon:		(20min AMRAP)						
Run			800m		AMRAP			Run
Front Rack KB Squats (32/20, 24/16)	(205/135, 155/95)		20		AMRAP			Squat (Front Rack KB)
Ring Rows			20		AMRAP			Ring Rows
Sit-ups			20		AMRAP			Sit-ups
Day 44								
Strength:								
Bench Press								Bench Press
70%			5		1			
80%			5		1			
90%			5		1			
100%			5		1			
110%			5		1			
MetCon:								
Power Clean (185/125, 135/85)	(185/125, 135/85)		10		4			Clean (Power)
Bar Facing Burpees	Bar Facing		10		4		3	Burpees (Bar Facing)
Auxiliary:								
DB Curls			21-15-9				4	Curls (DB)
DB Lateral Raises			21-15-9				1	Lateral Raises (DB)
Day 45								
Strength:								
Clean + Pause Jerk			2		6		5	Clean and Jerk
MetCon:								
HSPU			21-15-9					HSPU
Pull-ups			21-15-9					Pull-ups
Auxiliary:								
Row			100m		10			Row
Day 46								
Strength:								
Squat @ 82.5%	@ 82.5%		3		6			Squat
MetCon:								
Box Jumps			20		2			Box Jumps
Run			400m		2			Run
No Rest								
DL (245/165, 205/145)	(245/165, 205/145)		20		2			Deadlift

	Weights (lbs)	Time (min)	Reps	Distance	Sets		Sums	NOTES
Run			400m		2			Run
Day 47								
Strength:								
Hang Snatch @ 65-75%	@ 65-75%		2		4			Snatch (Hang)
Snatch @ 75-85%	@ 75-85%		1		4			Snatch
MetCon:								
DU's			50		3			DU's
KB Swings (32/24, 24/20)	(32/24, 24/20)		35		3			KB Swings
Elevated Push-ups	Elevated		25		3			Push-ups
Day 48								
Strength:								
Sumo DL @75%	@75%		5		5			Deadlift (Sumo)
MetCon:								
Row			1000m		1			Row
S2OH (165/105, 115/75)	(165/105, 115/75)		3,6,9,12,15		Ascending Ladder			Shoulder 2 Over-Head (S2OH)
T2B			15,12,9,6,3		cending x Descending Ladder			T2B
Auxiliary:								
Back Extensions			10		4		5	Back Extensions
Day 49								
Strength:								
Front Squat								Squat (Front)
65%			5		1			
75%			5		1			
80%			5		1			
85%			5		1			
90%			5		1			
MetCon:		(10min. AMRAP)						
Run			200m		AMRAP			Run
Goblet Hold Walking Lunges (32/24, 24/20)	(32/24, 24/20)		20paces		AMRAP			Lunges (KB Walking)
Burpees			10		AMRAP			Burpees
Day 50								
Strength:								
OHS Technique Work								Squat (Overhead)
w/ PVC			10		2			
w/ empty bar			10		2			
w/ @ 25%			8		2			
w/ @ 50%			4		2			
W/ @ 65-75%			2		2			
MetCon:								
Wall Balls			10		4			Wall Balls
Ring Rows (w/ feet elevated)	(w/ feet elevated)		15		4			Ring Rows
Bowtie sit-ups (35/25, 30/20)	(35/25, 30/20)		20		4			Sit-ups (Bowtie)
Day 51								
Strength:								
Bench Press								Bench Press
60%			5		1			
65%			5		1			
70%			5		1			
75%			5		2			
MetCon:								
For time w/ 15min. Cap								
Thrusters(75/45)	(75/45)		10		1			Thrusters
			10		1			Burpees
Burpee Box Jumps			10		1			Box Jumps
Thrusters (95/65)	(95/65)		8		1			Thrusters
			8		1			Burpees
Burpee Box Jumps			8		1			Box Jumps
Thrusters (115/85)	(115/85)		6		1			Thrusters
			6		1			Burpees
Burpee Box Jumps			6		1			Box Jumps
Thrusters (135/105)	(135/105)		4		1			Thrusters
			4		1			Burpees
			4		1			Burpees
Burpee Box Jumps			4		1			Box Jumps
Day 52								
Strength:								
Snatch + OHS + Hang Snatch			1		6			Snatch
			1					Snatch (Overhead)
			1					Snatch (Hang)
MetCon:								
Power Snatch (115/75, 85/55)	(115/75, 85/55)		9		4			Snatch (Power)
T2B			12		4			T2B
Run			300m		4			Run
Auxiliary:								
Loaded Back Extensions	Loaded		8		4			Back Extensions
Plank Holds			45-60sec		4			Plank Holds
Day 53								
Strength:								
Establish 5 RM Front Squat			?		?			Squat (Front)
MetCon:								
Wall Balls			30		2			Wall Balls

	Weights (lbs)	Time (min)	Reps	Distance	Sets		Sums	NOTES
KB Swings (32/24, 24/20)	(32/24, 24/20)		25		2			KB Swings
HRPU			20		2			HRPU
Strict Chin-ups			15		2			Chin-ups (Strict)
Auxiliary:								
Row @75%	@75%		1600m		1			Row
Day 54								
Strength:								
Rope Climbs			?		?			Rope Climbs
MetCon:								
			1		6			Squat (Overhead)
			1		6			Snatch (Hang)
Squat Cleans (185/125, 135/85)	(185/125, 135/85)		7		4			Squat Clean
S2OH			9		4			Shoulder 2 Over-Head (S2OH)
Auxiliary:								
DB Curls			10		1			Curls (DB)
Ring Dips			10		1			Ring Dips
Day 55								
Strength:		(8min. EMOM)						
DL (start @ 60%)	(start @ 60%)		3		1			Deadlift
65%			3		1 (@2min)			
70%			3		1 (@4min)			
75%			3		1 (@6min)			
MetCon:								
Pull-ups			25		1			Pull-ups
Burpees			10		1			Burpees
Run			400m		1			Run
Pull-ups			18		1			Pull-ups
Burpees			10		1			Burpees
Run			200m		1			Run
Pull-ups			11		1			Pull-ups
Burpees			10		1			Burpees
Run			100m		1			Run
Day 56								
Strength:								
Squat @80%	@80%		2		6			Squat
MetCon:								
DU's			100		2			DU's
SA KB Swings (32/20, 24/16)	(32/20, 24/16)		75		2			KB Swings (single)
Sit-ups			50		2			Sit-ups
Power Cleans (185/125, 135/85)	(185/125, 135/85)		25		2			Clean (Power)
Day 57								
Strength:								
KB Front Rack Walking Lunges	KB		16paces		3			Lunges (Front Rack Walking)
BB Front Rack Walking Lunges	BB		10paces		3		3	Lunges (Front Rack Walking)
MetCon:								
Run			800m		1			Run
Strict Chin-ups			5		5			Chin-ups (Strict)
Ring Push-ups			10		5			Push-ups (Ring)
Run			800m		1			Run
Day 58								
Strength:								
Establish a 5RM Bench Press								Bench Press
MetCon:								
KB Swings (32/24, 24/20)	(32/24, 24/20)		50-40-30-20-10					KB Swings
DU's			50-40-30-20-10					DU's
Every Minute drop and do 5 push-ups			every minute					Push-ups
Auxiliary:								
TGU			2each		5		0	TGU (KB)
Day 59								
Strength:		(7min. EMOM)						
Power Cleans (205/135, 155/105)	(205/135, 155/105)		5		Odd Minute			Clean (Power)
Bar Facing Burpees	Bar Facing		5		Even Minute			Burpees (Bar Facing)
MetCon:								
DL (225/155, 185/125)	(225/155, 185/125)		21-15-9					Deadlift
Pull-ups			21-15-9					Pull-ups
Day 60								
Strength:								
Squat @82.5%	@82.5%		2		6			Squat
MetCon:								
S2OH (135/85, 95/65)	(135/85, 95/65)		15		2			Shoulder 2 Over-Head (S2OH)
OH Walking Lunge			10paces		2			Lunges (OH Walking)
No rest								
S2OH (155/105, 115/85)	(155/105, 115/85)		10		2			Shoulder 2 Over-Head (S2OH)
Front Rack Waling lunges			10paces		2			Lunges (Front Rack Walking)
Day 61								
Strength:								

	Weights (lbs)	Time (min)	Reps	Distance	Sets		Sums	NOTES
Snatch @ 70-85%	@70-85%		2		6			Snatch
MetCon:		For Time						
Wall Balls	(30/20, 20/14)		40		1			Wall Balls
Run			400m		1			Run
HRPU			30		1			HRPU
Run			300m					Run
HSPU			20					HSPU
Run			200m				1	Run
Pistols (or heavy loaded step ups)			10each					Squat (Pistol)
Run			100m					Run
Auxilliary:								
DB Curls			12		4			Curls (DB)
Ring Dips			12		4			Ring Dips
Day 62								
Strength:								
DL @60%	@60%		5		2			Deadlift
70%			4		2			
75%			3		2			
MetCon:								
Row			350m		3			Row
Sit-ups			25		3			Sit-ups
KB Snatch (32/20, 24/16)	(32/20, 24/16)		20		3			Snatch (KB)
Goblet Squat			15		3			Squat (KB Goblet)
Auxiliary:								
Farmer Walks			100m		4		2	Farmer Walks
Day 63								
Strength:								
Jerks @ 70-75%	@ 70-75%		3		2			Jerk
75-80%	75-80%		2		2			
95-90%	95-90%		1		3			
MetCon:								
Muscle Ups			3,6,9					Muscle Ups
Power Cleans (205/140, 185/125)	(205/140, 185/125)		3,6,9					Clean (Power)
No Rest								
Strict Chin-ups					6,9,12			Chin-ups (Strict)
DU's			50		6,9,12			DU's
No Rest								
Burpees					9,12,15			Burpees
Run			400m		9,12,15			Run
Auxiliary:								
Prowler Push			100m		4		2	Prowler Push
Day 64								
Strength:		(10min. EMOM)						
Push-ups			10		Odd Minute (5)			Push-ups
Bent BB Rows			10		Even Minute (5)			Bent Row (BB)
MetCon:		(10min. AMRAP)						
DB Step-ups (35/25, 30/20)	(35/25, 30/20)		10		AMRAP			Step-ups (DB)
T2B			10		AMRAP			T2B
Ring Dips			10		AMRAP			Ring Dips
Day 65								
Strength:								
Establish 1RM Thruster								Thrusters
MetCon:								
Hang Power Clean (135/85, 95/65)	(135/85, 95/65)		9		3			Clean (Hang Power)
Thrusters (135/85, 95/65)	(135/85, 95/65)		15		3			Thrusters
Auxiliary:								
Row			250m		8			Row
Day 66								
Strength:								
20min to establish 1RM Back Squat	20min to establish 1RM							Squat
MetCon:		(20min time cap)						
DU's			100		1			DU's
KB Swings			80		1			KB Swings
Feet elevated push-ups	Feet elevated		60		1			Push-ups
pull-ups			40		1			Pull-ups
Single arm OH KB walking Lunges			20paces		1			Lunges (OH Walking, Single)
Running man singles			100		1			Running man single-unders
Day 67								
Strength:								
Snatch Pulls + Snatch			2		8			Snatch Pull
			1					Snatch
MetCon:								
8min. Ladder	8min. Ladder							
Front Squat (155/100, 115/85)	(155/100, 115/85)		3,6,9,12,…		8min			Squat (Front)
Box Jumps			3,6,9,12…		8min			Box Jumps
HSPU								HSPU
Auxiliary:								
Floor Sweeps			20		3			Floor Sweeps
Loaded Back Extensions	Loaded		10		3			Back Extensions

	Weights (lbs)	Time (min)	Reps	Distance	Sets		Sums	NOTES
Day 68								
No W.O.D. Holiday								
Day 69								
Strength:								
Bench Press @75-100% of BW	@75-100% of BW		10, 8, 6, 4, 2		5			Bench Press
Bent Double KB or DB Rows			8		4		1	Bent Row (KB/DB)
MetCon:								
Wall Balls			25		3			Wall Balls
Sit-ups			20		3			Sit-ups
Burpees			15		3			Burpees
Auxiliary:								
Sledge Strikes			20		3		4	Sledge Strikes
KB Cross Walks (switch hands every 50m)	(switch hands every 50m)		100m		4			Cross Walks (KB)
Day 70								
Strength:								
Power Clean + Front Squat + Jerk			1		5			Clean (Power) and Jerk
MetCon:								
HSPU			7		7			HSPU
Thrusters (135/95)	(135/95)		7		7			Thrusters
HSPU			7		7			HSPU
T2B			7		7			T2B
DL(245/175)	(245/175)		7		7			Deadlift
Burpees			7		7			Burpees
KB Swing (32/20)	(32/20)		7		7			KB Swings
Pull-ups			7		7			Pull-ups
Day 71								
Strength:								
KB Front Rack Elevated Split Squats			5each		5			Squat (Split, Front Rack KB)
MetCon:								
Run			300m		4			Run
Ball Slams			15		4			Ball Slams
Farmer Carry (32/20, 24/16)	(32/20, 24/16)		100m		4		1	Farmer Carry
Day 72								
Strength:								
Snatch			2		6			Snatch
MetCon:								
			2		5			Squat (Front)
Power Cleans (205/135, 155/95)	(205/135, 155/95)		6		5			Clean (Power)
Box Jumps			9		5			Box Jumps
HRPU			12		5			HRPU
Auxiliary:								
Row (Max Effort)	Max Effort		30sec On/30Ssec OFF		6			Row
Day 73								
Strength:								
Squats @80%	@80%		2		6			Squat
MetCon:								
DL (135/85, 95/65)	(135/85, 95/65)		15		1			Deadlift
S2OH			15		1			Shoulder 2 Over-Head (S2OH)
DU's			50		1			DU's
Rest			1min		1			
DL (135/85, 95/65)	(135/85, 95/65)		12		2			Deadlift
S2OH			12		2			Shoulder 2 Over-Head (S2OH)
DU's			35		2			DU's
Rest			2min		1			
DL (135/85, 95/65)	(135/85, 95/65)		10		3			Deadlift
S2OH			10		3			Shoulder 2 Over-Head (S2OH)
DU's			20		3			DU's
Day 74								
Strength:								
Clean			2		5			Clean
MetCon:		(15min. Time Cap)						
Pull-ups			21-15-9		3			Pull-ups
SA KB Swing (32/20, 24/16) reps are per arm	(32/20, 24/16)		21-15-9		3			KB Swings (single)
Row			1000m					Row
Day 75								
Strength:								
DL								Deadlift
60%			5		1			
70%			4		1			
80%			3		1			
85%			2		3			
MetCon:		(20min. AMRAP)						
Wall Balls			50		AMRAP			Wall Balls
Run			400m		AMRAP			Run
Sit-ups w/WB			40		AMRAP		1	Sit-ups (WB)
Run			300m		AMRAP			Run
Burpees			30		AMRAP			Burpees

	Weights (lbs)	Time (min)	Reps	Distance	Sets		Sums	NOTES
Run			200m		AMRAP			Run
Thrusters (135/85, 95/65)	(135/85, 95/65)		20		AMRAP			Thrusters
Run			100m		AMRAP			Run
Day 76								
Strength:								
Renegade Rows			10		4			Row (Renegade)
MetCon:								
Strict HSPU			15-12-9		3			HSPU (Strict)
KB/DB front Rack lunges (32/20, 24/16) per le	(32/20, 24/16) per leg		15-12-9		3		1	Lunges (KB/DB Front Rack)
T2B			15-12-9		3			T2B
Auxiliary:								
DB Curls			10		4			Curls (DB)
Band Tricep Extensions			20		4			Tricep Extensions (Band)
Day 77								
Strength:		10min. EMOM						
Front Squat Starting at 60%	Starting at 60%		?		10			Squat (Front)
(increase weight every round)	(increase weight every round)							
MetCon:								
Power Cleans (185/125, 135/95)	(185/125, 135/95)		AMRAP		3			Clean (Power)
Front Squats			AMRAP		3			Squat (Front)
Strict Chin-ups			AMRAP		3			Chin-ups (Strict)
KB Swing (32/24, 24/20)	(32/24, 24/20)		AMRAP		3			KB Swings
Row			AMRAP		3			Row
Day 78								
Strength:								
Bench Press	@70%		8		1			Bench Press
	@75%		8		1			
	@80%		4		3			
MetCon:								
Burpee Box Jumps	(30/24, 24/20)		5		4			Burpee Box Jumps
DB Alternating Press			10each		4			Press (DB)
Ring Rows			15		4			Ring Rows
Auxiliary:								
SLRDL			8each		4			RDL (single leg)
Day 79								
Strength:								
Clean and Jerk @70-85%	@70-85%		2		3			Clean and Jerk
MetCon:		(for time)						
Row			500m		1			Row
Air Squats			40		1			Squat (Air)
Sit-ups			30		1			Sit-ups
Push-ups			20		1			Push-ups
Pull-ups			10		1			Pull-ups
Auxiliary:								
DU's			3		max			DU's
Day 80								
Strength:								
Squat @80%	@80%		4		6			Squat
MetCon:		(13.4 - 7min. Ladder 3,6,9,12,15..)						
C&J (135/95)	(135/95)							Clean and Jerk
T2B								T2B
Auxiliary:								
Loaded Back Extensions	Loaded		8		4			Back Extensions
Day 81								
Strength:								
Hang Power Snatch + Hang Snatch + Snatch	(work up to 80%)		1		6			Snatch (Hang Power)
			1		6			Snatch (Hang)
			1		6			Snatch
MetCon:		25min cap						
Front Squat (185/125, 135/105)	(185/125, 135/105)		6		5			Squat (Front)
Burpees			12		5			Burpees
KB Swings (32/24, 24/20)	(32/24, 24/20)		18		5			KB Swings
Run			400m		5			Run
Day 82								
Strength:								
Deadlifts	@50%		8					Deadlift
	@55-60%		6					
	@65-70%		4					
	@75-80%		2					
	@85-90%		2					
MetCon:								
DL	(275/200, 225/186)		7		3			Deadlift
Strict Chin-ups			7		3			Chin-ups (Strict)
Deficit HSPU	one plate per side		7		3			HSPU (Deficit)
Auxiliary:								
Floor Sweeps			20		4			Floor Sweeps

	Weights (lbs)	Time (min)	Reps	Distance	Sets		Sums	NOTES
Day 83								
Strength:								
Bench Press starts @70% (increasing by 5%	starts @70% (increasing by 5% each round)		5		5			Bench Press
Standing One Arm KB/DB row			5each		5		1	Standing Row (KB/DB, single)
MetCon:								
DU's			100		1			DU's
Push-ups			20		4			Push-ups
OH Plate Walking Lunges (45/35)	(45/35)		20paces		4			Lunges (OH Walking)
Ring Rows			20		4			Ring Rows
DU's			100		1			DU's
Auxiliary:								
Heavy BB Curls			10		4			Curls (BB)
Ring Tricep Extensions			10		4			Tricep Extensions (Ring)
Day 84								
Strength:								
Squat @80%	@80%		2		6			Squat
MetCon:								
Row			3000m		1			Row
DB Thrusters (30/20, 20/14)	(30/20, 20/14)		150		1		2	Thrusters (DB)
Bowtie sit-ups (35/20, 25/20)	(35/20, 25/20)		120		1			Sit-ups (Bowtie)
Pull-ups			90		1			Pull-ups
Auxiliary:								
Loaded Back Extensions	Loaded		10		4			Back Extensions
Day 85								
Prowler Push			100m		10			Prowler Push
KB Farmer Walks/Rack Hold Walks	AHAP		100m		10		2	Farmer Walks (KB)
Overhead Sledge Strikes			10each		10			Overhead Sledge Strikes
Run			300m		10			Run
Auxiliary:								
Row			2000m		1			Row
Day 86								
Strength:								
Snatch			1		5			Snatch
C & J			1		5			Clean and Jerk
MetCon:								
Power Clean (185/125, 135/95)	(185/125, 135/95)		21,15,9		3			Clean (Power)
Bar Facing Burpees			21,15,9		3			Burpees (Bar Facing)
Auxiliary:								
Anti-rotational press			8each		4			Press (Anti-rotational)
Day 87								
Strength:								
Squats @80%	@80%		5		6			Squat
MetCon: 18min Cap		18min Cap						
HSPU			4		3			HSPU
Run			100m		3			Run
Pull-ups			8		3			Pull-ups
Run			100m		3			Run
HRPU			12		3			HRPU
Run			100m		3			Run
Air Squats			16		3			Squat (Air)
Run			100m		3			Run
Sit-ups			20		3			Sit-ups
Run			100m		3			Run
Day 88								
Strength:								
Hang Clean + Pause Jerk			2		6			Clean (Hang)
			2		6			Jerk (Pause)
MetCon:								
Thrusters (155/95, 115/75)	(155/95, 115/75)		10		3			Thrusters
KB Swings (32/24, 24/20)	(32/24, 24/20)		25		3			KB Swings
Auxilliary:								
Row			250m		4			Row
Day 89								
Strength:								
DL @80%	@80%		3		5			Deadlift
MetCon:								
Box Jumps (30/24, 24/20)	(30/24, 24/20)		10		4			Box Jumps
T2B			12		4			T2B
Row			15 Calories?		4			Row
Day 90								
Strength:								
Bench Press (10min EMOM)	(10min EMOM) 10-1 @ 75-100% of BW							Bench Press
MetCon: 90 second intervals		90 second intervals						
Wall Balls (20/14)	(20/14)		15		4			Wall Balls

	Weights (lbs)	Time (min)	Reps	Distance	Sets		Sums	NOTES
Strict Chin-ups			7		4			Chin-ups (Strict)
DU's			AMRAP		4			DU's
Auxiliary:								
Bar Roll-outs			10		4			Bar Roll-outs
Floor Sweeps			16		4			Floor Sweeps
Day 91								
Strength:								
OHS			3		5			Squat (Overhead)
MetCon:								
Rope Climbs			10		2			Rope Climbs
KB Walking lunge in Single arm Rack Hold (32	(32/20, 24/16)		50		2			Lunges (KB Walking, Single, Rack Hold)
KB Jerks			50		2		1	Jerk (KB)
Run			10		2			Run
Auxiliary:								
Sledge Strikes			20		4			Sledge Strikes
Med Ball Russian Twist	Med Balls		20		4		3	Russian Twists
Day 92								
Strength:								
Strict Press + Push Press + Split Jerk			1		6		1	Press (Strict)
			1		6			Push Press
			1		6			Split Jerk
MetCon:								
Row			500m		1			Row
Burpees			9		4			Burpees
Ball Slams			15		4			Ball Slams
Sit-ups with Slam Ball			21		4			Sit-ups
Row			500m		1			Row
Auxiliary:								
Ring Support Holds			20sec		4			Ring Support Holds
Ring Dips			10-Aug		4			Ring Dips
Day 93								
Strength: 7min EMOM		7min EMOM						
C & J@ 70-75%	@ 70-75%		2		7			Clean and Jerk
MetCon:								
Front Squats (205/135, 155/105)	(205/135, 155/105)		6		5			Squat (Front)
C2B Pull-ups			6		5			Pull-ups (C2B)
Auxiliary:								
Band Wood Chops			10each		4			Wood Chops (Band)
Day 94								
Strength:								
Squats @80%	@80%		2		6			Squat
MetCon:								
Run			400m		2			Run
HSPU			15		2			HSPU
Run			400m		2			Run
HRPU			15		2			HRPU
Day 95								
Strength:								
3 Position Snatch	3 Position							Snatch
MetCon:								
DU's			50		1			DU's
KB Swings (32/24, 24/20)	(32/24, 24/20)		50		1			KB Swings
Sit-ups			50		1			Sit-ups
DU's			25		1			DU's
KB Swings (32/24, 24/20)	(32/24, 24/20)		25		1			KB Swings
Sit-ups			25		1			Sit-ups
Auxiliary:								
Rope Pulls			10		1			Rope Pulls
Day 96								
Strength:								
Deadlifts								Deadlift
60%			5		1			
70%			4		1			
80%			3		1			
85%			2		1			
80%			3		1			
70%			4		1			
60%			5		1			
MetCon:								
Row			1500m		1			Row
			21		4			Ball Slams
Wall Balls (30/20, 20/14)	(30/20, 20/14)		25		3			Wall Balls
Ring Rows			20		3			Ring Rows
Goblet Hold Walking Lunge (32/24, 24/20)	(32/24, 24/20)		15paces		3		3	Lunges (KB Walking)
Day 97								
Strength:								
Establish a 3RM Push Press	3RM							Push Press
MetCon: 18min cap		(18min cap)						

	Weights (lbs)	Time (min)	Reps	Distance	Sets		Sums	NOTES
Squat Clean (165/115, 135/85)	(165/115, 135/85)		5		1			Squat Clean
Squat Clean (185/125, 155/105)	(185/125, 155/105)		3		1			Squat Clean
Squat Clean (225/150, 185/125)	(225/150, 185/125)		1		1			Squat Clean
T2B			40		1			T2B
			20		1			Burpees
Burpee Box Jumps			20		1			Box Jumps
Auxiliary:								
Med Ball Russian Twists	Med Balls		25		4			Russian Twists
Day 98								
Strength:								
Rope Climbing Skillz			15min		1			Rope Climbs
MetCon:								
Run Relay			400m		4		3	Run (Relay)
Thrusters (115/75)	(115/75)		21		1			Thrusters
Rope Climbs			12		1			Rope Climbs
Thrusters			15		1			Thrusters
Rope Climbs			9		1			Rope Climbs
Thrusters			9		1			Thrusters
Rope CLimbs			6		1			Rope Climbs
Run Relay			400m		4			Run (Relay)
Day 99								
Strength:								
DB Bench Press			8		5			Bench Press (DB)
SA DB/KB Bench Row			8each		5		1	Bench Row (DB/KB, single)
MetCon:								
Row			250m		4			Row
SA KB Swing (32/20, 24/16)	(32/20, 24/16)		30		4			KB Swings (single)
KB Goblet Hold Step-ups (20")	(20")		20		4			Step-ups (KB)
Day 100								
Strength:								
C & J @80%	@80%		2		5			Clean and Jerk
MetCon:			21-15-9					
S2OH	(135/85, 95/65)		21-15-9					Shoulder 2 Over-Head (S2OH)
Pull-ups			21-15-9					Pull-ups
Rest	3 minutes							
Front Squat	(185/125, 135/85)		15-9-6					Squat (Front)
Feet elevated Push-ups	Feet elevated		15-9-6					Push-ups
Auxilliary:								
Anti-rotational Press			8each		4			Press (Anti-rotational)
Day 101								
Strength:								
Squats @ 80%	@ 80%		6		4			Squat
MetCon:	(26min cap)							
Run Relay			200m		8			Run (Relay)
OHS (135/95, 115/75)	(135/95, 115/75)		50		1			Squat (Overhead)
Sit-ups			100		1			Sit-ups
DU's			150		1			DU's
Power Cleans			50		1			Clean (Power)
Box Jump overs (24/20)	(24/20)		100		1		1	Box Jump Overs
Day 102								
Strength:								
Snatch Catch w/2 sec. pause in bottom +Full S	w/2 sec. pause in bottom		2		5		1	Snatch (Catch)
			1					Snatch
MetCon:								
Row			500m		3			Row
Burpees			15		3			Burpees
KB SA Swing (24/16)	(24/16)		30		3			KB Swings (single)
Bar Roll Outs			10		4			Bar Roll-outs
Day 103								
Strength:								
Speed DL @70%	@70%		5		6		1	Deadlift (Speed)
MetCon:								
HSPU			7		5			HSPU
C2B Chin-ups			9		5		1	Chin-ups (C2B)
Day 104								
Strength: 10min. EMOM		10min. EMOM						
Bench Press (75-100% of BW)	(75-100% of BW)							Bench Press
MetCon: 10min AMRAP	(10min AMRAP)	(10min AMRAP)						
Power Cleans (185/125, 135/95)	(185/125, 135/95)		10		AMRAP			Clean (Power)
Wall Balls			15		AMRAP			Wall Balls
DU's			25		AMRAP			DU's
Auxiliary:								
Russian Twists			25		4			Russian Twists
Day 105								

	Weights (lbs)	Time (min)	Reps	Distance	Sets		Sums	NOTES
Strength:								
Front Squat (starting at 65% increasing load b	(starting at 65% increasing load by 5% each round)		5		5			Squat (Front)
MetCon:								
Run			400m		4			Run
Double KB Jerks (32/20, 24/16)	(32/20, 24/16)		12		4			Jerk (Double Bell KB)
KB Walking Lunges			12		4			Lunges (KB Walking)
Push-ups w/hands on handles of the bells	w/hands on handles of the bells		12		4			Push-ups
Auxiliary:								
Sledge Strikes			20		4			Sledge Strikes
TGU			3each		4			TGU (KB)
Day 106								
Strength:								
RDL			8		4			RDL
Bench KB/DB rows			8each		4		1	Bench Row (KB/DB)
MetCon:								
Ring Dips			10		3			Ring Dips
T2B			15		3			T2B
Ball Slams (30/20, 20/15)	(30/20, 20/15)		20		3			Ball Slams
Day 107								
Strength:								
Hang Power Clean + Hang Clean + Front Squat+ Jerk			1		5			Clean (Hang Power)
MetCon:								
Thrusters (135/85, 95/65)	(135/85, 95/65)		15		3			Thrusters
Lateral Burpees over the Bar			10		3			Burpees (Lateral Bar Over)
Run			400m		3			Run
Auxiliary:								
Floor Sweeps			10		4			Floor Sweeps
Loaded Glute Bridge	Loaded		10		4			Glute Bridge
Day 108								
Strength:								
Squats @85%	@85%		5		5			Squat
MetCon:								
Pull-ups			15-9-6-9-15		5			Pull-ups
Feet Elevated Push-ups	Feet Elevated (using 2 x 45lb plates)		15-9-6-9-15		5			Push-ups
DU's			15-9-6-9-15		5			DU's
Auxiliary:								
Row			500m		4			Row
Day 109								
Strength:								
Pause Snatch (working up to 80-85%)	(working up to 80-85%)		2		6			Snatch (Pause)
MetCon:	(25min cap)							
Row			1600m		1			Row
Hang Power Cleans Pos. #2 (185/125, 135/95	Pos. #2 (185/125, 135/95)		25		1			Clean (Hang Power)
Box Jumps			25		1			Box Jumps
							3	
Front Rack DB (2) Walking Lunges (40/20, 35,	(40/20, 35/25)		2		1			Lunges (Walking DB Front Rack)
Wall Balls (30/20, 20/14)	(30/20, 20/14)		25		1			Wall Balls
			25					Wall Balls
Wall Ball Sit-ups			25					Sit-ups
Day 110								
Strength:								
DL (starting @ 65%, increasing load by 5% ea	(starting @ 65%, increasing load by 5% each round)		2		5			Deadlift
MetCon:								
DL (225/155)	(225/155)		21-15-9		3			Deadlift
HSPU			21-15-9		3			HSPU
Day 111								
Strength:								
Bench Press								
60% of BW	60% of BW		10		1			Bench Press
70%			8		1			
80%			6		1			
90%			4		1			
100%			2		1			
Firebreather Bench press @ BW			10		1			Bench Press
@110%			8		1			
@120%			6		1			
@130%			4		1			
@140%			2		1			
MetCon:	(12min AMRAP)							

	Weights (lbs)	Time (min)	Reps	Distance	Sets		Sums	NOTES
Squat Cleans (205/135, 165/115)	(205/135, 165/115)		6		AMRAP			Squat Clean
T2B			12		AMRAP			T2B
KB Swings (32/24, 24/20)	(32/24, 24/20)		18		AMRAP			KB Swings
Auxiliary:								
Run			1600m		1			Run
Day 112								
Strength:								
OHS (As heavy as possible with perfect form.	AHAP		3		6			Squat (Overhead)
MetCon:								
S2OH (155/95, 105/75)	(155/95, 105/75)		9		4			Shoulder 2 Over-Head (S2OH)
Box Jumps			15		4			Box Jumps
Run			200m		4			Run
Auxiliary:								
Farmer Walks			100m		4			Farmer Walks
Sledge Strikes			20		4			Sledge Strikes
